# A CRISPR New World: Attitudes in the Public toward Innovations in Human Genetic Modification

**DOI:** 10.3389/fpubh.2017.00117

**Published:** 2017-05-22

**Authors:** Steven M. Weisberg, Daniel Badgio, Anjan Chatterjee

**Affiliations:** ^1^Department of Neurology, Center for Cognitive Neuroscience, University of Pennsylvania, Philadelphia, PA, USA

**Keywords:** genetic modification, online survey, Mechanical Turk, metaphor, CRISPR

## Abstract

The potential to genetically modify human germlines has reached a critical tipping point with recent applications of CRISPR-Cas9. Even as researchers, clinicians, and ethicists weigh the scientific and ethical repercussions of these advances, we know virtually nothing about public attitudes on the topic. Understanding such attitudes will be critical to determining the degree of broad support there might be for any public policy or regulation developed for genetic modification research. To fill this gap, we gave an online survey to a large (2,493 subjects) and diverse sample of Americans. Respondents supported genetic modification research, although demographic variables influenced these attitudes—conservatives, women, African-Americans, and older respondents, while supportive, were more cautious than liberals, men, other ethnicities, and younger respondents. Support was also was slightly muted when the risks (unanticipated mutations and possibility of eugenics) were made explicit. The information about genetic modification was also presented as contrasting vignettes, using one of five frames: genetic editing, engineering, hacking, modification, or surgery. Despite the fact that the media and academic use of frames describing the technology varies, these frames did not influence people’s attitudes. These data contribute a current snapshot of public attitudes to inform policy with regard to human genetic modification.

## Introduction

We are in the midst of a scientific revolution that will transform biological research and have profound effects on medicine (1–4). Recently, genetic modification using CRISPR-Cas9 (clustered regularly interspaced short palindromic repeat–CRISPR-associated protein), a system of adaptive immunity discovered in bacteria, has become widely feasible and cheap. By some estimates, it is 150 times cheaper than other genetic modification techniques such as the use of Zinc fingers, costing as little as $30 (5). Described in 2015 as “Science’s breakthrough of the year” (6), CRISPR-Cas9 heralds promise as well as perils. We might be able to eliminate single-gene disorders such as cystic fibrosis, hemophilia, and Huntington’s disease. At the same time, conscious selection of genes for specific physical and mental traits might reify social inequities and revive the possibility of eugenics.

With the first report of human germline modification in 2015 (7), these concerns take on an unprecedented urgency. The effects of these genetic modifications would be heritable and propagated into future generations. Researchers and ethicists are divided on how best to proceed (8, 9). Some call for a moratorium on human germline research since these techniques need to be refined, and more importantly, we need to understand their ethical implications and establish a communal understanding of how best to proceed (10–12). Others insist that we have a moral imperative to press on; the potential benefits to many combined with the observation that people are typically inept at anticipating social consequences of new technologies undermine reasons to wait (13–15). High profile media outlet opinions range from calls for caution to breathless anticipation (16, 17–21).

In December 2015, the US National Academies of Sciences, Engineering and Medicine held a summit on the regulation of CRISPR-Cas9 gene-modifying technology (22). The summit was opened by PHD physicist and Congressman Bill Foster (D-IL) with a reminder that gaining public acceptance of what scientists and physician want to do with CRISPR-Cas9 is critical (22). The subsequent discussions included the need for basic and preclinical research, issues around somatic and germline uses of CRISPR-Cas9, and the desire for an ongoing international forum for discussions that include a wide range of stakeholders including public interest advocates and members of the general public (23). The final report opined that it would be irresponsible to proceed with germline modification without broad societal consensus about the appropriateness of proposed applications (23).

These initial discussions underscore the need for information about the *public’s opinion* about genetic modification. Given CRISPR-Cas9’s technical ease, low cost, and potentially wide-spread application, knowing current public attitudes is critical to planning where to direct educational efforts and how to inform policy and regulations (24, 25).

Here, we conducted a large-scale survey of attitudes toward research made possible by CRISPR-Cas9. Our objective was to learn what level of support there is for this research among laypersons and to understand the variables that influence opinions regarding the ethics of genetic modification. Such initial information would serve as a yardstick of public opinion as debates continue among academics and the force of those controversies is communicated to the public. In this study, we tested two general hypotheses. First, the public’s reaction is not monolithic; social and demographic factors influence how people weigh *pros and cons* of such innovation (26). Second, the way information about new technology is presented influences people’s attitudes.

The social and demographic variables we considered were gender, age, ethnicity, education, and political affiliation. As expressed in a *Nature* comment, a 2015 biotechnology and ethical imagination summit (BEINGS 2015) gave the impression of gender differences with men focused on biosecurity threats and regulatory impediments to research and women on eugenics and the promotion of class, race, and gender inequities (27). We anticipated that younger people are more open to adopting new technologies. For example, age had an effect in a large survey conducted by *Nature* on people’s attitudes toward pharmacological enhancements in healthy individuals (28). Race and ethnicity may also affect people’s attitudes toward science (29, 30). Given the US history of unethical biomedical research on African-Americans, most infamously the US Public Health Service syphilis study at Tuskegee (31), it would not be surprising if this community were more sensitive to potential abuses of scientific research. Finally, political affiliations correlate with people’s attitudes toward complicated science (29). For example, conservatives are less likely than liberals to believe data supporting concerns about climate change (32, 33).

With respect to presentation of information, we considered two variables. We assessed the effects of explicit mention of risks of genetic modification, which we expect would dampen enthusiasm. We also assessed the effects of framing of the issue, which can bias people’s interpretation of complex events (34). The framing of an issue, often by the media, directs the public not only at what to think but also at how to think about an issue. When smoking was framed as a story about individual choice, it was unlikely to mobilize public support for tobacco regulation guidelines. However, when framed as a defective product that required the government to protect citizens, tobacco regulation gained support (35). Such framing can set up expectations as new information comes in that is configured to the defining frame. We used metaphors to frame research in genetic modification. Metaphors can influence people’s opinions about complex social issues and influence their views on policy. For example, when crime is discussed as a predator, people advocate for stronger policing; when crime is described as a virus, people advocate for cures for social ills that give rise to crime (36) [although see Ref. (37)].

To assess the effect of framing metaphors, we used contrasting vignettes (38, 39). This technique probes attitudes to minimally contrasting vignettes, using a between-subject design. Each participant is randomly assigned to a vignette and is unaware of contrasting versions. In addition to assessing people’s general attitudes, the method captures differences in attitudes rendered by differences in presentation. Each vignette is worded similarly, except for the framing and language supporting that framing. Each participant offers an opinion on one version of the vignettes. Our principal question was whether framing inﬂuences people’s attitudes at a population level.

In summary, we used contrasting vignettes to assess the public’s attitudes toward new innovations in genetic modification. We examined effects of how information is presented and the sensitivity of these attitudes to demographic variables. An understanding of the public’s view of genetic modification is critical to public outreach and to guiding policy on the pace and direction of this research, which will have a profound effect on all our lives.

## Materials and Methods

These studies were carried out in accordance with the recommendations of the IRB board of the University of Pennsylvania, with written informed consent from all subjects. All subjects gave written informed consent in accordance with the Declaration of Helsinki. The protocol #806447 was approved by the IRB board of the University of Pennsylvania.

### Metaphor Selection

**Figure 1:**
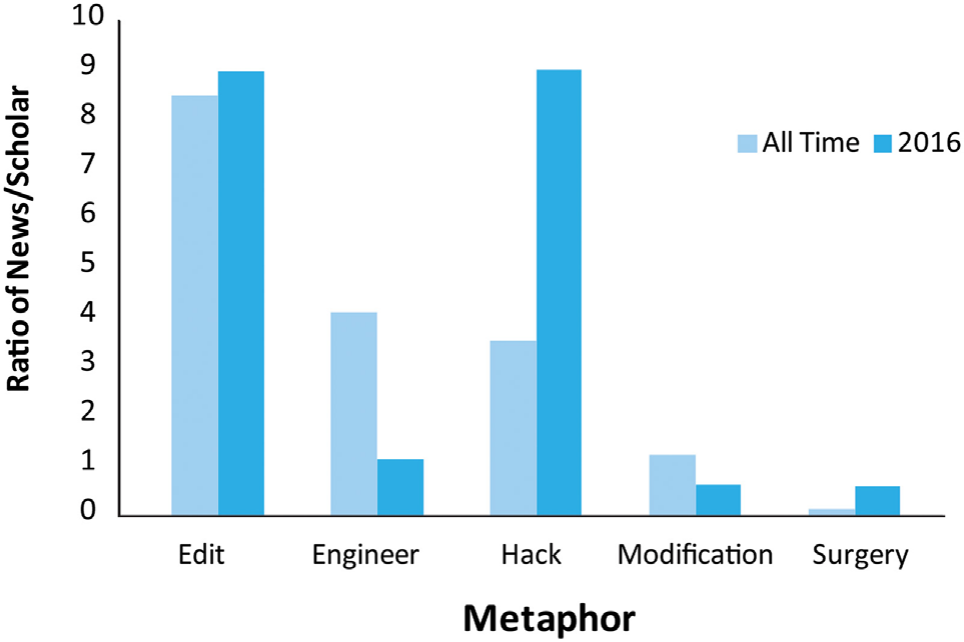
**Metaphor use in Google News and Google Scholar search results**. The ratio of hits found on Google News compared to Google Scholar for all time (light blue bars) and for 2016 (dark blue bars). The metaphors used to describe genetic modification appear on the *X*-axis. Hits were obtained by searching Google News or Google Scholar using (“gene [metaphor]” OR “genetic [metaphor]”). Then, the ratio of News/Scholar was obtained and graphed. Journalists publish proportionally more articles using the editing metaphor. All other metaphors appear more often in academic writing or equally frequently. See Table 1 for the raw numbers of hits from Google News and Google Scholar (obtained from Google searches on 12/19/2016).

We used *genetic modification* as a basic description and *genetic editing, genetic engineering, genetic surgery*, and *genetic hacking* as metaphors of interest. These metaphors were chosen because of their wide use in the academic literature and the popular press (see Table 1 and Figure 1). Unlike the crime as predator or crime as illness framing metaphors, we did not have clear predictions of how these metaphors might influence attitudes. We sought to get an impression of how scientists and journalists use these metaphors by examining the ratio of hits on Google News over Google Scholar. (Results were obtained by searches on 12/19/2016, in private browsing mode to ensure that browsing history and location were not taken into account by Google’s search algorithms, with queries of “genetic [metaphor]” OR “gene [metaphor].”) From this anecdotal examination, scientists and journalists seem to emphasize different metaphors in describing genetic modification. Scientists prefer to use modification and engineering, while journalists prefer editing. Conversely, scientists used editing relatively sparingly, whereas journalists used engineering and modification sparingly. Both groups seldom used hacking or surgery. Given these general differences in use of framing metaphors, the influence of language used by the press might have an influence on how the public interprets this research that is not intended by scientists.

**Table 1:** **Hits (and percentages) on Google News and Google Scholar for uses of various metaphors to describe genetic modification**.

Google hits	Editing	Engineering	Hacking	Modification	Surgery
**All time**					
News	91,800 (48.79%)	73,900 (39.28%)	92 (0.04%)	22,200 (11.80%)	157 (0.08%)
Scholar	10,800 (22.34%)	18,000 (37.23%)	26 (0.05%)	18,200 (37.76%)	1,320 (2.73%)
**2016**					
News	31,400 (62.20%)	15,200 (30.11%)	18 (0.04%)	3,830 (7.59%)	31 (0.06%)
Scholar	3,500 (15.10%)	13,400 (57.82%)	2 (0.00%)	6,220 (26.84%)	52 (0.22%)

*Raw number of hits (and percentages) from Google News and Google Scholar all time and in 2016 alone. Results were obtained by searching Google for (“genetic [metaphor]” OR “gene [metaphor]”)*.

### Study 1

#### Conditions

**Figure 2:**
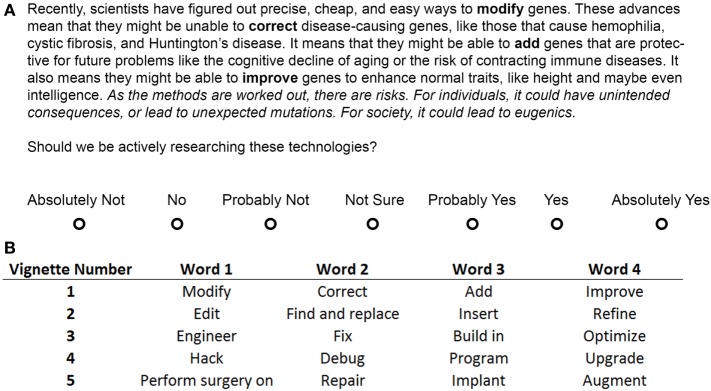
**Genetic modification vignette**. The vignette shown to participants in the Modify + Risk condition from Study 1 **(A)**. The Likert scale was displayed after the vignette had been on the screen by itself for 30 s. Words in bold were replaced by the corresponding words in the table **(B)** for participants in the other metaphor conditions. The words in italics were placed after the first sentence for the Study 2 Risk-before condition and were removed for the No Risk condition in Study 1. Bold and italic fonts are for emphasis only and were not seen by participants. See Supplementary Material for all vignettes for both studies in full.

We designed 10 vignettes by crossing two variables: risk (explicitly mentioned or not) and metaphor (Modify/Neutral, Edit, Engineer, Hack, Surgery). The vignette skeleton is shown in Figure 2. The bolded terms in the paragraph were replaced by the metaphoric terms in the lower portion of Figure 2. The sentences in italics were the explicitly mentioned risks, which were omitted in the non-explicit condition. Each vignette described three possible uses of genetic modification: the eradication of single-gene disorders, insertion of protective genes, and insertion or replacement of genes for enhancement. Explicit risks mentioned unintended individual consequences and the possibility of societal eugenics. Participants were randomly assigned to one of the 10 conditions in a between-participants design.

#### Participants

Using a power analysis based on data from Fitz and colleagues (39), we estimated needing 125 participants per condition and recruited 1,250 participants using Amazon’s Mechanical Turk (M-Turk). Amazon’s M-Turk (www.mturk.com) is an online tool, which allows researchers to collect data from users in exchange for monetary compensation. Users receive ratings from administrators on studies they complete, allowing requesters to ensure that their sample is reliable. The study was called “Opinions about science,” and paid $0.25. We restricted participants to people in the US who had completed greater than 500 M-Turk studies and had an approval rate greater than 95%. We discarded one subject whose ID could not be verified on M-Turk. The final number of participants used in analyses was 1,249.

#### Procedure

In order to participate, participants first agreed to waive documentation of informed consent by clicking “Accept” on M-Turk. Written consent was not obtained to maintain participant confidentiality, and avoid storing identified data. The waiver was made available to participants as a PDF, as well as contact information for the lab. This procedure was approved by the IRB. After providing informed consent, participants were instructed that they would be reading a paragraph about recent developments in science, after which they would be asked their opinion. Participants were told that a paragraph would appear for 30 s. After 30 s, a button appeared. They could then continue reading, or advance to the question by clicking the button, taking as much time as they wished. Once they advanced, a question appeared below the vignette: “Should we be actively be researching these technologies?” Responses were indicated by selecting: Absolutely Not; No; Probably Not; Not Sure; Probably Yes; Yes; and Absolutely Yes. Participants had as much time to respond as they wanted. Finally, participants entered demographic information, including age, gender, years of education, political affiliation, and whether they or someone they knew had a genetic condition (and, if yes, what the condition was). We coded political affiliation as described in Table 2. The frequencies are reported along with the other demographic variables in Table 3 alongside percentages in the American public according to the American Census and the American Community Survey.

**Table 2:** **Coding scheme for political affiliation**.

Coding	Participant responses
Left	Communist, D, Dem, Democrat, Left, Liberal, Progressive, Socialist
Right	Conservative, GOP, R, Republican, Rep, Right, Tea Party
Independent	Independent, Independent leaning [Democrat/Republican]
Moderate	Moderate
Blank	None, no affiliation, neutral, N/A, neither, unaffiliated, [no text]
Other	Green, libertarian, anarchist, [other]

*Common responses (separated by commas) given by participants to the question of their political affiliation, and how those responses were coded. Participant responses are non-exhaustive. See Data Sheets S1 and S2 in Supplementary Material to view actual participant responses*.

**Table 3:** **Demographic variables by percentage**.

Demographics	Study 1 (*n* = 1,249)	Study 2 (*n* = 1,244)	United States (*n* = 323,127,513)
**Gender**			
Males	56.5	49.5	49.2
Females	43.5	50.5	50.8
**Ethnicity**			
Asian	8.0	7.5	5.6
Black/African-American	6.3	6.9	13.3
Hispanic/Latino	5.1	5.9	17.6
White	80.6	79.8	61.6
**Politics**			
Left	43.2	40.2	48
Right	14.0	17.4	39
Independent	21.1	19.2	13
Moderate	2.2	3.0	
Others	2.9	4.1	6
Blank	16.7	16.1	
**Education**			
High school or less	12.0	13.4	41.5
Some college	31.3	31.0	31.3
4 years of college	35.0	32.0	17.4
>4 years of college	21.7	23.7	9.8
Age [mean(SD)]	33.23 (12.29)	35.20 (11.75)	Median = 37.2

*We omitted 10 participants total who did not wish to report gender. We also omitted three categories of ethnicities, which constituted less than 3% (American Indian/Alaskan Native; Native Hawaiian or Pacific Islander; Other; and Do Not Wish to Say). US population statistics (except Politics) obtained using most recent available data on 1/6/2017 from www.census.gov and the American Community Survey. Statistics for Politics obtained from Pew Research Center (http://www.people-press.org/2015/04/07/2014-party-identification-detailed-tables/). We used “lean Democrat” and “lean Republican” to match our coding of participant responses as closely as possible*.

### Study 2

#### Conditions

We used the same metaphors from Study 1, but all participants now read vignettes that included the sentence about risks. In Study 1, in the Risk condition, the risks were explicitly mentioned at the end of the vignette. In Study 2, for half the participants, the risks were presented as the second sentence in the vignette, before the metaphor (Risk-before); for the other half, the risks were presented at the end, after the metaphor as in Study 1 (Risk-after). Since explicit mention of risks had a dampening effect, we wished to learn whether leading with risks rather than mentioning them after the potential uses of genetic modification would further modify people’s attitudes.

#### Participants

We recruited 1,250 participants using Amazon’s M-Turk. The study was called “Opinions about science,” and paid $0.25. We restricted participants to those who were located in the US, had completed greater than 500 M-Turk studies, and who had an approval rate greater than 95%. In this study, we also restricted participation to those who had not participated in Study 1. We discarded six subjects whose IDs could not be verified on M-Turk. The final number of participants used in analyses was 1,244.

#### Procedure

The procedure was identical to Study 1.

## Results

### Public Attitudes and Demographics Analyses

In Study 1, participants were supportive of research into genetic modification. Comparing the distribution of all participants’ responses (−3 = Absolutely Not, to 3 = Absolutely Yes) to a neutral attitude (Not Sure = 0) reveals strong support (M = 1.65, SD = 1.32) and a large effect size, one-sample *t*(1,248) = 44.23, *p* < 0.0001, *d* = 2.50. Because of the non-normality of the distribution of responses (skewed toward ceiling), we also ran a one-sample Kolmogorov–Smirnov test and obtained similar results (*D* = 0.205, *p* < 0.0001).

In Study 2, participants were also supportive of genetic modification research (M = 1.38, SD = 1.46) one-sample *t*(1,243) = 33.30, *p* < 0.0001, *d* = 1.89. Again, data were not normally distributed, but a one-sample Kolmogorov–Smirnov test revealed similar results (*D* = 0.183, *p* < 0.0001).

See Table 3 for the percentages of participants tabulated for gender, ethnicity, politics, and education. None of the demographic variables interacted with Metaphor, Risk, or Study, so we combined data from both studies to analyze the effects of demographic variables for a total of 2,493 participants.

We found a significant effect of gender, *t*(2,481) = 8.50, *p* < 0.0001, *d* = 0.34, such that men were more supportive of proceeding with research (M = 1.74, SD = 1.31) than women (M = 1.27, SD = 1.45). We found a significant effect of ethnicity (only including ethnicities representing more than 5% of the total data—Asian, Black/African-American, Hispanic/Latino, and White), *F*(3, 2,413) = 11.95, *p* < 0.0001, ω^2^ = 0.013. Follow-up pairwise contrasts revealed this effect was driven by African-American participants supporting genetic modification research less enthusiastically than other ethnicities (all *p*’s < 0.0001), while none of the other ethnicities differed (all *p*’s > 0.18). We found a significant effect of political affiliation, *F*(5, 2,487) = 15.68, *p* < 0.0001, ω^2^ = 0.03. Follow-up Bonferroni-corrected pairwise contrasts, with a threshold of α < 0.0033, revealed that Right respondents (M = 1.72, SD = 1.26) supported genetic modification research significantly less often than Left respondents (M = 1.06, SD = 1.53). Independent respondents (M = 1.63, SD = 1.39) followed a similar pattern to Left respondents, supporting genetic modification significantly more than Right respondents. We found a significant effect of education, coded categorically as in Table 3, *F*(3, 2,489) = 3.581, *p* = 0.013, ω^2^ = 0.003. This effect was driven by participants with a high school degree or less responding less favorably (M = 1.29, SD = 1.46) than participants with some college (M = 1.51, SD = 1.50), 4 years of college (M = 1.56, SD = 1.36), and more than 4 years of college (M = 1.58, SD = 1.26). We observed a significant effect of age, *r*(*n* = 2,493) = −0.09, *p* < 0.0001, such that older participants were less supportive of human genetic modification research than younger participants. Finally, we did not observe an effect for whether participants (*n* = 206) had a genetic disease, or were related to someone with a genetic disease, *t*(2,491) = 0.35, *p* = 0.71, *d* = 0.01.

### Metaphor and Risk Analyses

#### Study 1

To determine whether being explicit about possible risks and the metaphor used to describe genetic modification influenced participant attitudes toward research, we conducted a 2 (Risk or No Risk) by 5 (Metaphor condition) between-subjects ANOVA with response (−3 = Absolutely Not, to 3 = Absolutely Yes) as the dependent variable. We observed a main effect of Risk, *F*(1, 1,239) = 36.02, *p* = 0.0001, ω^2^ = 0.03, such that participants who read the vignettes that included risks were less supportive (M = 1.43, SD = 1.36) than participants who read the vignettes which did not include risks (M = 1.87, SD = 1.24). We did not observe an effect of Metaphor, *F*(4, 1,239) = 0.90, *p* = 0.47, ω^2^ = 0.0, and there was no interaction, *F*(4, 1,239) = 1.51, *p* = 0.33, ω^2^ = 0.0005.

**Figure 3:**
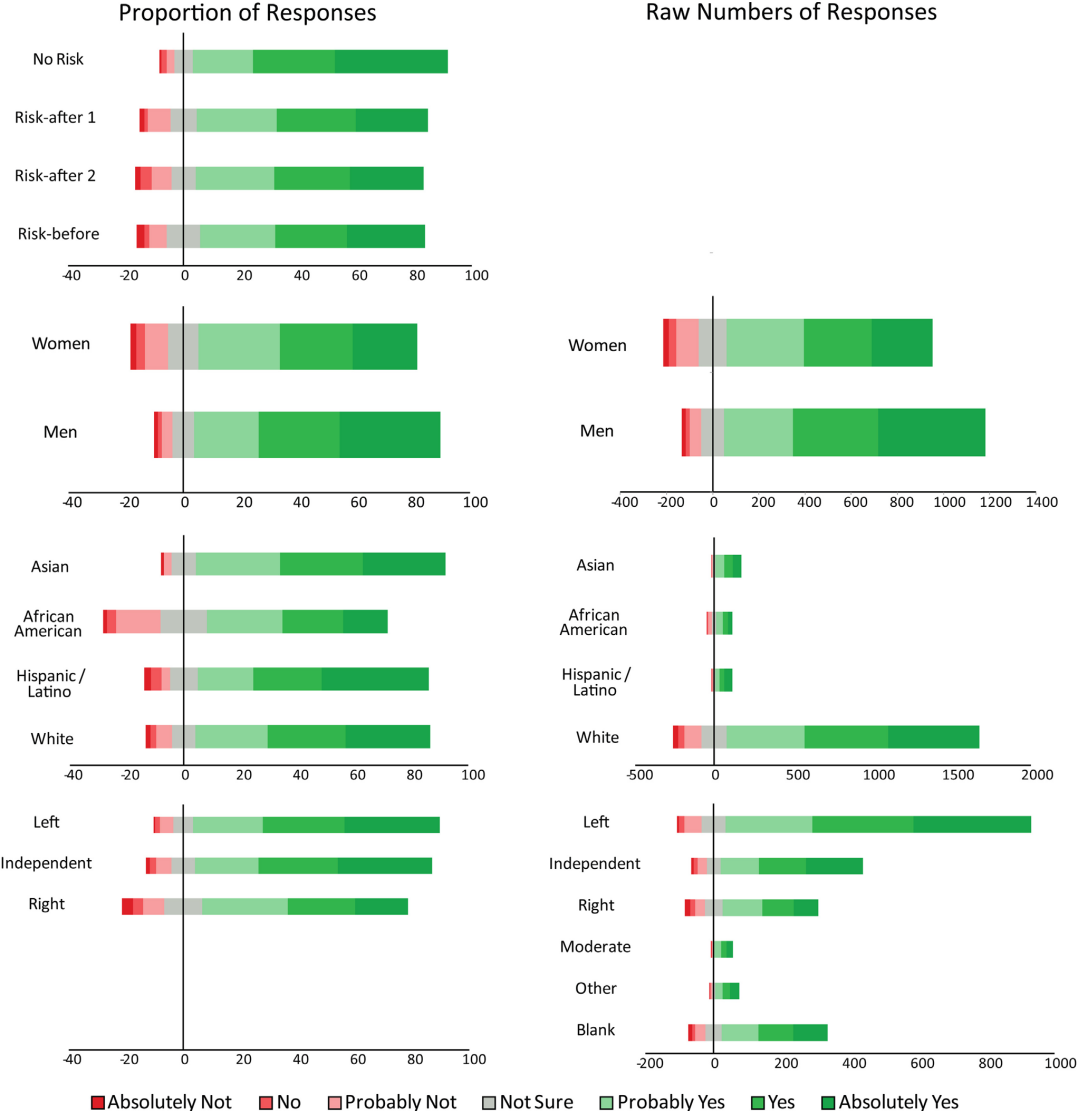
**Proportions and raw numbers of survey responses**. The proportions (left panel) and raw numbers (right panel) of responses to the question “Should we be actively be researching these technologies?” Data are presented so that positive (green) and negative (red) responses can be easily compared. Negative values represent negative responses. The top proportion graph is not reproduced in raw numbers, since participants were randomly and equally assigned into the No Risk, Risk-after (risks mentioned after vignette) Study 1, Risk-after Study 2, and Risk-before (risks mentioned before vignettes). The lower three graphs break down the responses by demographic variables—gender, ethnicities, and politics.

Figure 3 displays breakdowns of participant responses. The separate graphs sort the data using different parameters (e.g., the graphs that are second from the top break down the sample by gender). Each horizontal bar depicts the apportionment of responses for one subset of participants. The graph in the upper left breaks down responses by risk condition and the first two horizontal bars refer to the risk conditions in Study 1. Note that the vast majority of both bars are green, indicating that the majority of subjects responded positively. However, when risks are included, the proportion of the bar that is red increases, indicating that more participants responded less favorably.

#### Study 2

Again, we conducted a 2 (Risk-before or Risk-after) by 5 (Metaphor condition) between-subjects ANOVA with response (−3 = Absolutely Not, to 3 = Absolutely Yes) as the dependent variable. We observed no significant effects. There was no effect of Metaphor, *F*(1, 1,234) = 1.52, *p* = 0.20, ω^2^ = 0.002, no effect of Risk, *F*(4, 1,234) = 0.09, *p* = 0.77, ω^2^ = 0.0, and no interaction, *F*(4, 1,239) = 0.41, *p* = 0.81, ω^2^ = 0.0.

## Discussion

The technical feasibility and low cost of CRISPR-Cas9 is likely to make this technology used widely. It could allow medicine to eliminate single-gene disorders, insert protective genes, and potentially replace or modify genes to enhance physical and mental traits. While gene modification techniques have been available for a few decades, these latest innovations are likely game changers.

The use of CRISPR-Cas9 in human germlines reported in 2015 (7) has given scientists pause. Recent meetings have been organized to generate discussions about the future applications of these technologies. Scientists, ethicists, and the media express a wide range of opinions. Missing in these discussions is a sense of how the general public would react to both the promise and the perils of such technology. This paper represents a first step in filling this gap in knowledge as scientists and policymakers ponder how best to proceed.

The most basic finding, based on 2,493 respondents, is that our sample of diverse participants is supportive of continuing research in human genetic modification. Our vignettes gave respondents information about the possibility of eradicating single-gene disorders, inserting protective genes, and introduced the potential use of genetic modification for enhancement.

We further tested the following hypotheses. First, the form in which information is conveyed and social and demographic variables modify people’s opinions. With respect to the presentation of information, we found that the explicit mention of risks dampened enthusiasm for the use of such technologies. Despite this dampening effect, the respondents remained supportive of the research.

The relative support for pursuing this technology was modulated by several demographic variables. We think it would be a mistake to view people as pro- or anti-science, or pro- or anti-risk. Rather, we postulate that these demographic variables affect the relative weighting of potential promises and perils of this technology. Women are typically less optimistic about biotechnology than men (40). They may be more sensitive to risks and concerned about issues like eugenics, whereas men tend to be more concerned about regulatory impediments to scientific progress (27). Consistent with these speculations, we found that women as a group were less enthusiastic about proceeding with genetic modification. Similarly, African-Americans were less enthusiastic about this technology. Age also played a role. This observation is consistent with previous surveys that suggest that younger people are more open to the use of pharmacologic enhancements and are likely to be less averse to risks (28). Education and political affiliation affected peoples’ attitudes. People with a high school education or less were more cautious in their appraisal of the use of this technology. People with left-leaning politics were most positive about forging ahead. People with right-leaning politics, while still positive as a group, were more subdued in their endorsement, consistent with the observation that since 1974 there has been a steady diminution of trust in science (41) among conservatives.

We recognize that a limitation of this convenience sample is that our respondents, by virtue of taking an online survey, may be more open to promises of new technology. Other factors may have biased our sample, including an age distribution that skewed younger, and more left leaning in their political orientation compared to the general population. As we quantify in Table 3, it should be emphasized that the present sample also differs from the US population on proportion of African-Americans, Latinos, and Whites, and on proportion of highly educated people, over-representing Whites and the highly educated. We note that traditional sampling methods of using telephone calls have biases in so far as the use of landlines is on the decline. A recent non-peer reviewed report (42) surveying a representative sample of the US public also found general support for genetic manipulation, although not as robustly supportive as our group. The M-Turk population is nonetheless diverse and samples from a wide range of education and income levels (43).

We also tested the hypothesis that metaphors used to describe genetic modification bias people’s attitudes. Metaphors are important in guiding the general understanding of scientific advances (44) and are hypothesized to influence people’s views on the use of CRISPR-Cas9 (26). We examined the metaphors of editing, engineering, surgery, and hacking. One limitation of the current work is that the metaphors were chosen based on an *ad hoc* selection of metaphors which we observed commonly in academic and popular press writing. Although we did not find additional metaphors widely in use, it is possible that an inductive search for common metaphors would yield different results. We found anecdotal differences in the use of these framing metaphors between the academic literature and the media. Specifically, the media uses the editing metaphor more often than academics. Perhaps, journalists’ use of the editing as a core metaphor is not surprising, given their profession as writers.

The lay public needs to be informed of these dramatic biological innovations even as they are evolving. Scientific organizations call for engaging people from all sectors of society in a debate about genetic modification including the use of human embryos in this research (36). Such engagement is critical to understanding if there is broad public support for relevant health policies and regulations. However, engaging the public is predicated on educating them about new technologies, their real world implications, and establishing an initial measure of their attitudes toward the promise and perils of such research. The present data suggest that public education efforts have substantial influences on attitudes depending on whether the risks of genetic modification are made explicit.

We did not find that common metaphors used to describe genetic modification influenced people’s attitudes. From our data, there is no evidence that the media’s use of language influences people’s attitudes in ways that scientists might not intend. We remain agnostic as to whether the absence of an effect in this study represents a “dose response.” Perhaps, more detailed information and language supporting metaphors might modulate people’s attitudes (37). Alternatively, this sample of participants with a relatively high degree of support for research in genetic modification may have been at ceiling such that they were insensitive to modulation by framing metaphors.

## Conclusion

In summary, this survey of 2,493 Americans begins to fill a critical gap in knowledge. That is, what does the non-academic public think of genetic modification in humans now that it is feasible and cheap? Understanding the public’s attitudes is critical to education efforts and to formulating public policy. Here, we present a snapshot of attitudes in the US. We recognize that the public’s attitudes may evolve over time as more information is disseminated and people engage more fully with the issues at hand. Based on our vignettes and this sample of the US public, people seem supportive of research in genetic modification. The degree of this support is attenuated by several factors. Women, older people, African-Americans, and people with less education, and those with right-leaning politics, while still supportive, are less so, perhaps representing greater sensitivity to potential risks and unintended consequences of these technologies than men, younger people, White and Latino Americans, and people more education and with left-leaning politics.

## Ethics Statement

This study was carried out in accordance with the recommendations of the IRB board of the University of Pennsylvania, with written informed consent from all subjects. All subjects gave written informed consent in accordance with the Declaration of Helsinki. The protocol #806447 was approved by the IRB board of the University of Pennsylvania.

## Author Contributions

SW, AC, and DB designed and conceptualized the study. SW and DB developed the methods and materials and coded the data. SW conducted the analyses, created the figures and tables, and drafted the manuscript. AC edited the manuscript.

## Conflict of Interest Statement

The authors declare that the research was conducted in the absence of any commercial or financial relationships that could be construed as a potential conflict of interest.
