# Corrigendum: A CRISPR New World: Attitudes in the Public toward Innovations in Human Genetic Modification

**DOI:** 10.3389/fpubh.2017.00161

**Published:** 2017-07-06

**Authors:** Steven M. Weisberg, Daniel Badgio, Anjan Chatterjee

**Affiliations:** ^1^Department of Neurology, Center for Cognitive Neuroscience, University of Pennsylvania, Philadelphia, PA, United States

**Keywords:** genetic modification, online survey, Mechanical Turk, metaphor, CRISPR

Error in Figure/Table

In the original article, there was a mistake in Figure 2 as published. **The original Figure 2 contained a typo in Figure 2. The sentence “These advances mean that they might be *UNABLE”* should have read “These advances mean that they might be *ABLE*”. This typo was solely in the Figure. The correct version of the vignette was presented to participants**. The corrected Figure 2 appears below. The authors apologize for this error and state that this does not change the scientific conclusions of the article in any way.

**Figure 2:**
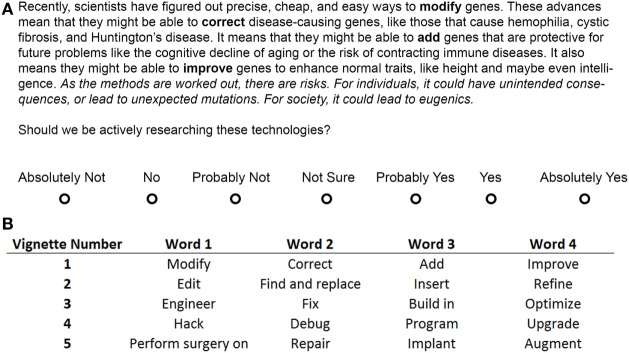
**Genetic modification vignette**. The vignette shown to participants in the Modify + Risk condition from Study 1 **(A)**. The Likert scale was displayed after the vignette had been on the screen by itself for 30 s. Words in bold were replaced by the corresponding words in the table **(B)** for participants in the other metaphor conditions. The words in italics were placed after the first sentence for the Study 2 Risk-before condition and were removed for the No Risk condition in Study 1. Bold and italic fonts are for emphasis only and were not seen by participants. See Supplementary Material for all vignettes for both studies in full.

## Conflict of Interest Statement

The authors declare that the research was conducted in the absence of any commercial or financial relationships that could be construed as a potential conflict of interest.

