# Supplementary material for: A CRISPR New World: Attitudes in the Public toward Innovations in Human Genetic Modification
Source: Front Public Health. 2017 May 22;5:117. doi: 10.3389/fpubh.2017.00117 (PMC5439143; doi:10.3389/fpubh.2017.00117)
Supplement: Supplementary file 1 [file Data_Sheet_1.DOCX]

**ON MECHANICAL TURK:**

**Instructions**

You are invited to participate in a research study being performed by researchers at the University of Pennsylvania Center for Cognitive Neuroscience. Your participation in this research study is voluntary and anonymous. You will be paid for your participation. You may decline further participation, at any time, without adverse consequences. This research study, 806447, has been approved by the University of Pennsylvania IRB.

If you are not a native English speaker, then you are not qualified to participate in this task.

CONTACT INFORMATION
This study is being conducted by the Center for Cognitive Neuroscience at the University of Pennsylvania located in philadelphus, Pa. If you have questions, please use the Contact Requester link to contact the requester.
You can also visit our website [here](http://ccn.upenn.edu/chatterjee/index.html) to learn more about our research.

If you have questions pertaining to your rights as a research participant, you may contact the Institutional Review Board (IRB) Office of the University of Pennsylvania, at 215.573.2540 or burgess4@upenn.edu.

BY CLICKING “ACCEPT”
[View Waiver of Documentation of Consent (PDF)](http://ccn.upenn.edu/chatterjee/anjan_pdfs/OnlineICF_MTurkWaiver_feb2015.pdf)

By accepting this HIT, you are confirming that you have read the Waiver of Documentation of Consent and agree to participate in this study.

Please click the link below to access the study:

[REDACTED]

At the end of the study, you will receive a confirmation code.

Please enter that confirmation code here:

**ON QUALTRICS**

Please enter your age:

Years of education (12 = Completed high school, +1 for each additional year):

Was English your first language?

- Yes (1)
- No (2)

Do you normally write with your RIGHT hand?

- Yes (1)
- No (2)

Our funding agency (National Institutes of Health) asks that we also obtain the following information from each participant so that NIH can monitor gender and minority inclusion in research studies.  Please check all that apply. Thank you.

What sex were you assigned at birth?

- Male (1)
- Female (2)
- Do not wish to say (3)

What is your gender?

- Male (1)
- Female (2)
- Transgender (3)
- Do not identify as male or female (gender non-conforming, gender queer) (4)
- Do not wish to say (5)

What is your sexual orientation?

- Lesbian, gay, or homosexual (1)
- Straight or heterosexual (2)
- Bisexual (3)
- Other--please specify (4) ____________________
- Do not wish to say (5)

Race/Ethnicity:

- American Indian / Alaska Native (1)
- Asian (2)
- Black or African American (3)
- Hispanic / Latino (4)
- Native Hawaiian or Pacific Islander (5)
- White (6)
- Other (7)
- Unknown (8)
- Do not wish to say (9)

Do you have any comments or feedback about the task?

These questions are not required, but help us get a more accurate picture of our data.

What is your political affiliation?

Do you or does anyone in your family have a genetic disorder?

- Yes (1)
- Not sure (2)
- No (3)

[If Yes is selected: ]

What is the genetic disorder?
