# Supplementary material for: A CRISPR New World: Attitudes in the Public toward Innovations in Human Genetic Modification
Source: Front Public Health. 2017 May 22;5:117. doi: 10.3389/fpubh.2017.00117 (PMC5439143; doi:10.3389/fpubh.2017.00117)
Supplement: Supplementary file 2 [file Data_Sheet_2.DOCX]

**Study 1 Vignettes**

1. Neutral + Risks

Recently, scientists have figured out precise, cheap, and easy ways to modify genes. These advances mean that they might be able to correct disease-causing genes, like those that cause hemophilia, cystic fibrosis, and Huntington’s disease. It means that they might be able to add genes that are protective for future problems like the cognitive decline of aging or the risk of contracting immune diseases. It also means they might be able to improve genes to enhance normal traits, like height and maybe even intelligence. Even as the methods are worked out, there are risks. For individuals, this could have unintended consequences, or lead to unexpected mutations. For society, it could lead to eugenics.

2. Neutral No Risks

Recently, scientists have figured out precise, cheap, and easy ways to modify genes. These advances mean that they might be able to correct disease-causing genes, like those that cause hemophilia, cystic fibrosis, and Huntington’s disease. It means that they might be able to add genes that are protective for future problems like the cognitive decline of aging or the risk of contracting immune diseases. It also means they might be able to improve genes to enhance normal traits, like height and maybe even intelligence.

3. Edit + Risks

Recently, scientists have figured out precise, cheap, and easy ways to edit genes. These advances mean that they might be able to find and replace disease-causing genes, like those that cause hemophilia, cystic fibrosis, and Huntington’s disease. It means that they might be able to insert genes that are protective for future problems like the cognitive decline of aging or the risk of contracting immune diseases. It also means they might be able to refine genes to enhance normal traits, like height and maybe even intelligence. Even as the methods are worked out, there are risks. For individuals, this could have unintended consequences, or lead to unexpected mutations. For society, it could lead to eugenics.

4. Edit No Risks

Recently, scientists have figured out precise, cheap, and easy ways to edit genes. These advances mean that they might be able to find and replace disease-causing genes, like those that cause hemophilia, cystic fibrosis, and Huntington’s disease. It means that they might be able to insert genes that are protective for future problems like the cognitive decline of aging or the risk of contracting immune diseases. It also means they might be able to refine genes to enhance normal traits, like height and maybe even intelligence.

5. Engineer + Risks

Recently, scientists have figured out precise, cheap, and easy ways to engineer genes. These advances mean that they might be able to fix disease-causing genes, like those that cause hemophilia, cystic fibrosis, and Huntington’s disease. It means that they might be able to build in genes that are protective for future problems like the cognitive decline of aging or the risk of contracting immune diseases. It also means they might be able to optimize genes to enhance normal traits, like height and maybe even intelligence. Even as the methods are worked out, there are risks. For individuals, this could have unintended consequences, or lead to unexpected mutations. For society, it could lead to eugenics.

6. Engineer No Risks

Recently, scientists have figured out precise, cheap, and easy ways to engineer genes. These advances mean that they might be able to fix disease-causing genes, like those that cause hemophilia, cystic fibrosis, and Huntington’s disease. It means that they might be able to build in genes that are protective for future problems like the cognitive decline of aging or the risk of contracting immune diseases. It also means they might be able to optimize genes to enhance normal traits, like height and maybe even intelligence.

7. Hack + Risks

Recently, scientists have figured out precise, cheap, and easy ways to hack genes. These advances mean that they might be able to debug disease-causing genes, like those that cause hemophilia, cystic fibrosis, and Huntington’s disease. It means that they might be able to program genes that are protective for future problems like the cognitive decline of aging or the risk of contracting immune diseases. It also means they might be able to upgrade genes to enhance normal traits, like height and maybe even intelligence. Even as the methods are worked out, there are risks. For individuals, this could have unintended consequences, or lead to unexpected mutations. For society, it could lead to eugenics.

8. Hack No Risks

Recently, scientists have figured out precise, cheap, and easy ways to hack genes. These advances mean that they might be able to debug disease-causing genes, like those that cause hemophilia, cystic fibrosis, and Huntington’s disease. It means that they might be able to program genes that are protective for future problems like the cognitive decline of aging or the risk of contracting immune diseases. It also means they might be able to upgrade genes to enhance normal traits, like height and maybe even intelligence.

9. Surgery + Risks

Recently, scientists have figured out precise, cheap, and easy ways to perform surgery on genes. These advances mean that they might be able to repair disease-causing genes, like those that cause hemophilia, cystic fibrosis, and Huntington’s disease. It means that they might be able to implant genes that are protective for future problems like the cognitive decline of aging or the risk of contracting immune diseases. It also means they might be able to augment genes to enhance normal traits, like height and maybe even intelligence. Even as the methods are worked out, there are risks. For individuals, this could have unintended consequences, or lead to unexpected mutations. For society, it could lead to eugenics.

10. Surgery No Risks

Recently, scientists have figured out precise, cheap, and easy ways to perform surgery on genes. These advances mean that they might be able to repair disease-causing genes, like those that cause hemophilia, cystic fibrosis, and Huntington’s disease. It means that they might be able to implant genes that are protective for future problems like the cognitive decline of aging or the risk of contracting immune diseases. It also means they might be able to augment genes to enhance normal traits, like height and maybe even intelligence.

**Study 2 Vignettes**

1. Neutral + Risks

Recently, scientists have figured out precise, cheap, and easy ways to modify genes. These advances mean that they might be able to correct disease-causing genes, like those that cause hemophilia, cystic fibrosis, and Huntington’s disease. It means that they might be able to add genes that are protective for future problems like the cognitive decline of aging or the risk of contracting immune diseases. It also means they might be able to improve genes to enhance normal traits, like height and maybe even intelligence. Even as the methods are worked out, there are risks. For individuals, this could have unintended consequences, or lead to unexpected mutations. For society, it could lead to eugenics.

2. Neutral Risks First

Recently, scientists have figured out precise, cheap, and easy ways to modify genes. Even as the methods are worked out, there are risks. For individuals, this could have unintended consequences, or lead to unexpected mutations. For society, it could lead to eugenics. These advances mean that they might be able to correct disease-causing genes, like those that cause hemophilia, cystic fibrosis, and Huntington’s disease. It means that they might be able to add genes that are protective for future problems like the cognitive decline of aging or the risk of contracting immune diseases. It also means they might be able to improve genes to enhance normal traits, like height and maybe even intelligence. 

3. Edit + Risks

Recently, scientists have figured out precise, cheap, and easy ways to edit genes. These advances mean that they might be able to find and replace disease-causing genes, like those that cause hemophilia, cystic fibrosis, and Huntington’s disease. It means that they might be able to insert genes that are protective for future problems like the cognitive decline of aging or the risk of contracting immune diseases. It also means they might be able to refine genes to enhance normal traits, like height and maybe even intelligence. Even as the methods are worked out, there are risks. For individuals, this could have unintended consequences, or lead to unexpected mutations. For society, it could lead to eugenics.

4. Edit Risks First

Recently, scientists have figured out precise, cheap, and easy ways to edit genes. Even as the methods are worked out, there are risks. For individuals, this could have unintended consequences, or lead to unexpected mutations. For society, it could lead to eugenics. These advances mean that they might be able to find and replace disease-causing genes, like those that cause hemophilia, cystic fibrosis, and Huntington’s disease. It means that they might be able to insert genes that are protective for future problems like the cognitive decline of aging or the risk of contracting immune diseases. It also means they might be able to refine genes to enhance normal traits, like height and maybe even intelligence.

5. Engineer + Risks

Recently, scientists have figured out precise, cheap, and easy ways to engineer genes. These advances mean that they might be able to fix disease-causing genes, like those that cause hemophilia, cystic fibrosis, and Huntington’s disease. It means that they might be able to build in genes that are protective for future problems like the cognitive decline of aging or the risk of contracting immune diseases. It also means they might be able to optimize genes to enhance normal traits, like height and maybe even intelligence. Even as the methods are worked out, there are risks. For individuals, this could have unintended consequences, or lead to unexpected mutations. For society, it could lead to eugenics.

6. Engineer Risks First

Recently, scientists have figured out precise, cheap, and easy ways to engineer genes. Even as the methods are worked out, there are risks. For individuals, this could have unintended consequences, or lead to unexpected mutations. For society, it could lead to eugenics. These advances mean that they might be able to fix disease-causing genes, like those that cause hemophilia, cystic fibrosis, and Huntington’s disease. It means that they might be able to build in genes that are protective for future problems like the cognitive decline of aging or the risk of contracting immune diseases. It also means they might be able to optimize genes to enhance normal traits, like height and maybe even intelligence.

7. Hack + Risks

Recently, scientists have figured out precise, cheap, and easy ways to hack genes. These advances mean that they might be able to debug disease-causing genes, like those that cause hemophilia, cystic fibrosis, and Huntington’s disease. It means that they might be able to program genes that are protective for future problems like the cognitive decline of aging or the risk of contracting immune diseases. It also means they might be able to upgrade genes to enhance normal traits, like height and maybe even intelligence. Even as the methods are worked out, there are risks. For individuals, this could have unintended consequences, or lead to unexpected mutations. For society, it could lead to eugenics.

8. Hack Risks First

Recently, scientists have figured out precise, cheap, and easy ways to hack genes. Even as the methods are worked out, there are risks. For individuals, this could have unintended consequences, or lead to unexpected mutations. For society, it could lead to eugenics. These advances mean that they might be able to debug disease-causing genes, like those that cause hemophilia, cystic fibrosis, and Huntington’s disease. It means that they might be able to program genes that are protective for future problems like the cognitive decline of aging or the risk of contracting immune diseases. It also means they might be able to upgrade genes to enhance normal traits, like height and maybe even intelligence.

9. Surgery + Risks

Recently, scientists have figured out precise, cheap, and easy ways to perform surgery on genes. These advances mean that they might be able to repair disease-causing genes, like those that cause hemophilia, cystic fibrosis, and Huntington’s disease. It means that they might be able to implant genes that are protective for future problems like the cognitive decline of aging or the risk of contracting immune diseases. It also means they might be able to augment genes to enhance normal traits, like height and maybe even intelligence. Even as the methods are worked out, there are risks. For individuals, this could have unintended consequences, or lead to unexpected mutations. For society, it could lead to eugenics.

10. Surgery Risks First

Recently, scientists have figured out precise, cheap, and easy ways to perform surgery on genes. Even as the methods are worked out, there are risks. For individuals, this could have unintended consequences, or lead to unexpected mutations. For society, it could lead to eugenics. These advances mean that they might be able to repair disease-causing genes, like those that cause hemophilia, cystic fibrosis, and Huntington’s disease. It means that they might be able to implant genes that are protective for future problems like the cognitive decline of aging or the risk of contracting immune diseases. It also means they might be able to augment genes to enhance normal traits, like height and maybe even intelligence.
